# Convective Drying of Papaya Seeds: Impact of Ethanol Pretreatment

**DOI:** 10.1111/1750-3841.70721

**Published:** 2025-11-24

**Authors:** Amanda Aparecida de Lima Santos, Maria Eduarda Teixeira, Paula Giarolla Silveira, Jefferson Luiz Gomes Corrêa

**Affiliations:** ^1^ Department of Food Science Federal University of Lavras Lavras Brazil; ^2^ Institute of Exact Sciences Federal University of Juiz de Fora Juiz de Fora Brazil

**Keywords:** by‐product valorization, combined resistance model, functional compounds, scanning electron microscopy

## Abstract

Papaya seeds are agroindustrial residues rich in bioactive compounds, lipids, and proteins. Their high moisture content makes them vulnerable to chemical and microbiological deterioration. Drying is a practical process to extend shelf life. The immersion in ethanol can be used as a drying pretreatment for improving the final quality and reducing drying time. This study examined the effect of ethanol on drying kinetics, specific energy consumption, cell‐wall thickness, quality parameters (water activity, color, phenolics, flavonoids, and antioxidant activity), oil yield, and mass‐transfer mechanisms via mathematical modeling. Seeds were immersed in ethanol for 2 min and convectively dried at 50°C or 70°C (air velocity 1 m s^−1^). The pretreatment shortened drying time by up to 55% and reduced energy demand by 54.07% at 50°C and 31.13% at 70°C, while inducing structural thinning of the cell wall that enhanced mass transfer. Drying preserved total phenolics and ABTS antioxidant activity, although flavonoids were more susceptible to ethanol‐related degradation. Lowering water activity proved effective for product stability, and drying at 50°C with ethanol offered better color retention. Mathematical modeling confirmed that ethanol altered mass‐transfer behavior, yielding a mixed regime governed by both internal diffusion and external convection. Ethanol pretreatment coupled with temperature control thus optimizes papaya‐seed drying, supporting their use as a sustainable ingredient for the food and pharmaceutical industries.

**Practical Applications:**

This study demonstrates that ethanol pretreatment prior to convective drying of papaya seeds is an effective strategy to reduce drying time and energy consumption while preserving functional properties such as phenolic compounds and antioxidant activity. The dried seeds exhibit microbiological stability and potential for use in functional foods, dietary supplements, and cosmetics. Moreover, the technique offers a sustainable solution for agro‐industrial waste valorization, contributing to circular economy practices and adding value to the papaya supply chain.

## 1 Introduction

Industrial fruit processing generates large volumes of residues, peels, seeds, and pulp, whose composition depends on the fruit processed (Bitencourt et al. 2022; de Souza et al. 2018). These by‐products, however, can be upgraded into value‐added ingredients, foods, supplements, and functional compounds, since non‐conventional fruit parts are rich in fiber, micro‐ and macronutrients, and exhibit antioxidant and anti‐inflammatory activity (Kowalska et al. 2017). Papaya (*Carica papaya L*.) is a tropical fruit of great economic importance (Cruz et al. 2025). Beyond fresh consumption, papaya is valued for papain, a proteolytic enzyme widely used in the food and pharmaceutical sectors (Chielle et al. 2016a). Its pulp is rich in vitamins A, B, and C, whereas latex, leaves, and seeds contain alkaloids (carpaine, pseudocarpaine) and benzyl isothiocyanate, compounds with antimicrobial and anthelmintic activity (Barroso et al. 2016).

Although usually discarded, papaya seeds (PS) contain up to 30% protein and 25%–30% lipids, making them promising sources of nutraceutical oils and protein ingredients (Kumoro et al. 2020). They also supply insoluble fiber and phenolic antioxidants that can replace synthetic preservatives and enhance oxidative stability in food matrices (Jiao et al. 2023; Shaheen and Farag 2023). Valorizing this co‐product therefore adds revenue, reduces waste, and helps achieve a zero‐waste papaya supply chain; yet their high moisture content demands rapid, well‐controlled drying to prevent enzymatic degradation and quality loss (Chielle et al. 2016b).

Drying, a ubiquitous preservation unit operation, removes moisture, extending food shelf life. Convective drying is the most used drying technique (Macedo et al. 2023; Silveira et al. 2024). It is a large time and energy demand process. Drying pretreatments are therefore employed to accelerate moisture removal, reduce energy use, and preserve functional properties and retain nutrients (Santos et al. 2024; Silveira et al. 2024).

Ethanol immersion has emerged as a safe and easy to apply drying pretreatment (Carvalho et al. 2020; Santos et al. 2024). The mixture of moisture‐ ethanol presents lower liquid‐phase surface tension and higher vapor pressure. Preferential evaporation of ethanol at the interface establishes composition and surface‐tension gradients that trigger the Marangoni effect, creating internal convective flows that drive residual moisture toward the surface and accelerate the drying process (Junqueira et al. 2021). Concurrently, partial solubilization of pectins and hemicelluloses can reduce cell‐wall thickness, lowering internal diffusion resistance and further enhancing mass transfer. Significant reductions in drying time and energy consumption have been reported for bananas, strawberries, and potatoes (Corrêa et al. 2012; Macedo et al. 2023); however, ethanol pretreatment (ET) has never been applied to PS, a gap addressed by this work.

The evaluation of the influence of ET on convective drying of PS, focusing on drying time, energy consumption, color parameters, phenolic and flavonoid contents, antioxidant activity, oil yield, and cell‐wall thickness was the goal of the present study. In addition, the prevailing mass‐transfer resistance (internal, external, or mixed) was determined through mathematical modeling.

## 2 Materials and Methods

### 2.1 Material

PS (*Carica papaya L., formosa cultivar)* were obtained from a local market (Lavras, MG state, Brazil). The proximate composition of fresh PS (Table 1) was determined according to AOAC (2019). Seeds were removed from the fruit cavities, washed to eliminate adhered pulp, placed on a sieve, and gently rubbed to detach the exotesta, a protective outer film covering the pores. They were then rinsed, packed in polyethylene bags, and frozen at −8°C until use, when they were thawed (De Lima Santos et al. 2024).

**TABLE 1:** Proximate composition and quantification of macrominerals and microminerals in papaya seeds.

Parameter (w.b.) (%)	Whole matter
Water content	69.619 ± 0.292
Ethereal extract	7.501 ± 0.369
Proteins	2.737 ± 0.082
Fibers	6.750 ± 0.344
Ash	1.533 ± 0.020
Non‐nitrogenous extract	11.860 ± 0.722

### 2.2 Experimental Conditions

For the pretreatment, PS were immersed in 95% ethanol for 2 min at a material‐to‐solvent proportion of 1:5 (w/w), following Macedo et al. (2021) and Santos et al. (2024). Both untreated (fresh) and ethanol‐pretreated samples were subsequently dried in a convective tunnel dryer (Eco Engenharia Educacional, model MD018, São José, Brazil) operating with forced airflow at 1 m s^−1^ and temperatures of 50°C or 70°C (Silveira et al. 2024). The experimental setup is presented in Table 2.

**TABLE 2:** Experimental design for papaya seeds convective drying.

Code	Temperature (°C)	Ethanol pretreatment
50	50	No
50ET	50	Yes
70	70	No
70ET	70	Yes

Abbreviation: ET, ethanol treatment.

For every run, 69.75 ± 0.29 g of PS were evenly spread in a single layer on a stainless‐steel support. The experimental device allowed the continuous measurement of the sample mass during the drying, every 5 min during the first hour of the experiment and, subsequently, every 10 min, which was performed until reaching a moisture content of 10.58 ± 0.31% (wet basis). Moisture ratio (MR) was calculated with Equation (1).

(1) $$\mathrm{MR}=\frac{X_{t}-X_{e}}{X_{o}-X_{e}}$$

where $X_{e}\;$ is the equilibrium moisture content (dry basis), $X_{t}$ is the moisture content at time t (dry basis), and $X_{o}$ is the initial moisture content (dry basis).

#### 2.2.1 Diffusional Approximation (Model 1)

Moisture migration in food matrices is primarily driven by internal diffusion and is commonly represented by Fick's second law (Crank 1975; D'Avila et al. 2020), as expressed in Equation (2):

(2) $$\frac{\partial X}{\partial t}=\frac{D_{\mathrm{eff}}}{r^{2}}\left[\frac{\partial }{\partial r}\left(r^{2}\frac{\partial X}{\partial r}\right)\right]$$

where X denotes the moisture content (dry basis) at time t (s), r is the radial position (m), and $D_{\mathrm{eff}}\;$ is the effective moisture diffusivity (m^2^ s^−1^).

To obtain a solution, Equation (2) was applied under the assumptions of (i) uniform initial distribution of water within the material (Equation 3), (ii) spherical geometry as a boundary condition (Equation 4), and (iii) negligible external mass‐transfer resistance (Equation 5), following the approach described by D'Avila et al. (2020).

(3) $$X\left(r,0\right)=X_{o}$$

(4) $$\frac{\partial X}{\partial r}\left(0,t\right)=0$$

(5) $$X\left(R,t\right)=X_{e}$$

where $X_{o}$ corresponds to the initial moisture conten (dry basis), $X_{e}$ to the equilibrium moisture content (dry basis), and R to the average particle radius (m).

Following Nguyen et al. (2023), for drying times > 5 min, only the first term of the series was retained (Equation 6). To account for deviations from a perfect sphere, a sphericity factor (φ) was applied, as proposed by Luisetti et al. (2023), replacing R with φ R in Equation (6).

(6) $$\begin{array}{c} \mathrm{MR}=\frac{6}{\pi^{2}}\sum_{n=1}^{\infty }\exp\left(-n^{2}\pi^{2}D_{\mathrm{ef}f_{1}}\frac{t}{{\left(R\right)}^{2}}\right)=\frac{6}{\pi^{2}}\exp\left(-\pi^{2}D_{\mathrm{ef}f_{1}}\frac{t}{{\left(\varphi R\right)}^{2}}\right) \end{array}$$

#### 2.2.2 Internal–External Resistance Approach (Model 2)

In most drying scenarios, mass transfer is not dictated exclusively by internal diffusion (Corrêa et al. 2017). Relying on a purely diffusive model may therefore lead to inaccurate interpretations. To distinguish the prevailing mechanism, Nguyen et al. (2023) suggested the use of the Biot number ($Bi_{m}$) (Equation 7), which expresses the ratio between internal and external resistances. When $Bi_{m}$ > 100, internal diffusion dominates; for $Bi_{m}$ < 0.1, the process is mainly governed by external resistance. Intermediate values (0.1 ≤ $Bi_{m}$ ≤ 100) indicate that both mechanisms play a role, resulting in a mixed‐control regime (Nguyen et al. 2023; Rurush et al. 2022).

(7) $$Bi_{m}=\frac{k_{p}L_{o}}{\alpha \rho_{s}D_{\mathrm{eff}}}$$

where $k_{p}$ represents the mass transfer coefficient on a vapor pressure basis (kg _water_ m^−2^ s^−1^ kPa^−1^); $L_{o}$ denotes the characteristic length (m); $\rho_{s}$ corresponds to the density of the dry solid (kg _dry solids_ m^−3^); $D_{\mathrm{eff}}\;$ is the effective moisture diffusivity (m^2^ s^−1^); and α is a correction factor that reflects the chemical potential difference between the phases (kg_water_ kg_dry solid_^−1^ kPa^−1^).

When the magnitudes of external and internal resistances are of the same order, the drying process occurs under a mixed‐control regime. By substituting the parameters defined in Equation (7) and applying standard simplifications, Equation (8) is obtained, representing the behavior of this combined resistance mechanism (Nguyen et al. 2023).

(8) $$\mathrm{MR}=\frac{2B{i_{m}}^{2}}{{\beta_{1}}^{2}\left({\beta_{1}}^{2}+B{i_{m}}^{2}+Bi_{m}\right)}\exp\left(-\frac{\beta_{1}D_{\mathrm{ef}f_{2}}}{R^{2}}t\right)$$

where $\beta_{1}$ represents a function of $Bi_{m}$, expressed by Equation (9).

(9) $$\beta_{1}=0.099+\frac{1.446Bi_{m}}{0.846+Bi_{m}}$$

In situations where moisture transfer is controlled exclusively by external resistance and the influence of internal diffusion can be disregarded, Equation (10) is employed.

(10) $$\mathrm{MR}=\exp\left(-\frac{k_{x}A}{\alpha m_{s}}t\right)$$

where A denotes the surface area (m^2^); α is the equilibrium ratio at the solid–air interface (kg_water_ kg_dry solid_^−1^ kPa^−1^); $m_{s}$ represents the dry‐matter concentration (kg_dry matter_ m^−3^); and $k_{x}$ is the convective mass‐transfer coefficient expressed on a dry‐basis moisture content (kg_water_ kPa^−1^ m^−2^ s^−1^). The value of $k_{x}$​ can be estimated using the correlation presented in Equation (11).

(11) $$k_{x}=Bk_{\mathrm{air}}{\left(\frac{\rho_{\mathrm{air}}}{\mu_{\mathrm{air}}}\right)}^{1/2}\upsilon^{1/2}Pr^{1/3}$$

B is a constant that embodies material properties, whereas $k_{\mathrm{air}}$, $\rho_{\mathrm{air}}$, $\mu_{\mathrm{air}}$, υ, and Pr denote, respectively, the thermal conductivity, density, dynamic viscosity, velocity, and Prandtl number of the drying air. As demonstrated by Nguyen et al. (2023), the coefficient $k_{x}\;$ is strongly influenced by airflow velocity, whereas temperature changes from 50°C to 60°C exert only a minor effect.

Regardless of whether moisture removal is dominated by internal diffusion, external resistance, or a combination of both, the drying kinetics can generally be described using the exponential form shown in Equation (12) (Nguyen et al. 2023):

(12) $$\mathrm{MR}=\frac{X_{t}-X_{e}}{X_{o}-X_{e}}=a\exp\left(-bt\right)$$

where the parameters a (dimensionless) and b (s^−1^) vary with the prevailing mass‐transfer regime; their values are listed in Table 3.

**TABLE 3:** Drying kinetics models under internal, mixed, and external mass transfer control.

Mass transfer control	MR	*a*	*b*	Equation
Internal	$\frac{6}{\pi^{2}}\exp\left(-\pi^{2}D_{\mathrm{ef}f_{1}}\frac{t}{R^{2}}\right)$	$\frac{6}{\pi^{2}}$	$\pi^{2}D_{\mathrm{ef}f_{1}}\frac{t}{R^{2}}$	(14)
Mixed	$\exp\left(\frac{0.7599Bi_{m}}{2.1+Bi_{m}}\right)\exp\left(-\frac{\beta_{1}D_{\mathrm{ef}f_{2}}}{R^{2}}t\right)$	$\exp(\frac{0.7599Bi_{m}}{2.1+Bi_{m}})$	$\frac{\beta_{1}D_{\mathrm{ef}f_{2}}}{R^{2}}$	(15)
External	$\exp(-\frac{k_{x}A}{\alpha m_{s}}t)$	1	$\frac{k_{x}A}{\alpha m_{s}}$	(16)

When the prevailing mechanism cannot be clearly identified, the mixed‐resistance approach can be applied to estimate both the $Bi_{m}$ and the effective diffusion coefficient $D_{\mathrm{ef}f_{2}}$. The relationship between the Biot parameter and the coefficient a is expressed in Equation (13) (Dincer and Dost 1996):

(13) $$a=\exp\left(\frac{0.7599Bi_{m}}{2.1+Bi_{m}}\right)$$

By plotting the moisture ratio against drying time, drying‐kinetics curves for PS were obtained. These experimental data were fitted to two mathematical models: the Fickian diffusion model (Equation 14, Model 1) and the mixed‐resistance model (Equation 15, Model 2). For comparison, the case of purely external control was also considered, described by Equation (16).

The drying kinetics of PS were obtained by plotting the moisture ratio as a function of drying time. To describe these data, two models were fitted: the Fick diffusion model (Equation 14, Model 1), widely employed in drying studies to represent internal mass transfer resistance, and the mixed‐resistance model (Equation 15, Model 2), proposed by Nguyen et al. (2023) to simultaneously account for both internal and external resistances. In addition, for comparison purposes, the solution representing exclusively the external resistance was also considered (Equation 16, Model 2).

Model fitting was performed in Statistica 8.0 using non‐linear regression with the Quasi‐Newton method. This algorithm approximates the Hessian matrix to iteratively update parameter estimates, ensuring faster convergence and stable solutions compared with simple gradient‐based methods. The Quasi‐Newton approach is widely applied in studies involving model fitting, including drying kinetics, due to its efficiency, robustness, and accuracy when estimating a limited number of parameters, as in the present study. Goodness of fit was assessed by the sum of squared errors (SSE), calculated with Equation (17):

(17) $$\mathrm{SSE}=\sum_{i=1}^{n}{\left(MR_{\exp}-MR_{\mathrm{pred}}\right)}^{2}$$

where MR_exp_ and MR_pred_ are the experimental and predicted moisture ratios, respectively.

#### 2.2.3 Energy Consumption

The energy required by the dryer ($E_{t}$) to process PS at each air‐temperature setting was calculated with Equation (18) (Corrêa et al. 2021):

(18) $$E_{t}=Av_{a}\rho_{a}tC_{a}\Delta t$$

where $E_{t}$ is the total energy consumed (kWh); A is the cross‐sectional area of the sample holder (m^2^); $v_{a}\;$ the air velocity (m s^−1^); $\rho_{a}$ the air density (kg m^−3^); *t* the total drying time (h); $C_{a}$ the specific heat of air (kJ kg^−1^ K^−1^); and Δt the air‐temperature rise across the heater (K).

The specific energy required to remove 1 kg of water was obtained with Equation (19) (Silveira et al. 2024):

(19) $$\mathrm{EC}=\frac{E_{t}}{m_{i}-m_{f}}$$

where $m_{i}$ and $m_{f}$ are the initial and final sample masses, respectively.

### 2.3 Sample Characterization

Fresh and dried seeds were evaluated for moisture content, water activity, sphericity, color, total phenolics, total flavonoids, and antioxidant activity. Table 4 lists the physical properties of the fresh material.

**TABLE 4:** Physical characterization of fresh papaya seed.

Parameter	Value
Longitudinal diameter (mm)	4.79 ± 0.32
Transversal diameter (mm)	3.68 ± 0.37
Thickness (mm)	3.13 ± 0.23
Sphericity	0.79 ± 0.01
*L* ^*^	15.15 ± 0.89
*a*	2.18 ± 0.26
*b*	9.25 ± 0.30
*C* ^*^	9.51 ± 0.33
*h* ^*^	76.82 ± 1.38
Water activity (*a*_w_)	0.979 ± 0.004

Moisture content was determined gravimetrically in a vacuum oven (Solab SL104/40, Piracicaba, Brazil) at 70°C until constant weight (AOAC 2019). Water activity (*a*_w_) was measured at 25°C with an electronic hygrometer (Aqualab 3‐TE, Decagon Devices, Pullman, WA, USA).

Sphericity (φ) was obtained from Equation (20), assuming isometric properties of a sphere. Three samples of 15 seeds each were measured with the digital caliper.

(20) $$\varphi =\frac{{\left(lw\mathrm{th}\right)}^{\frac{1}{3}}}{l}$$

where *l*, w, and th are seed length, width, and thickness, respectively.

The color of 15 seeds per replicate was recorded with a colorimeter (Konica Minolta CR‐400) in CIELAB space (*L* ^*^ *a* ^*^ *b* ^*^); chroma (*C* ^*^) and hue angle (*h* ^*^) were also calculated. The total color difference (Δ*E*) was obtained from Equation (21) (De Souza et al. 2022):

(21) $$\Delta E=\sqrt{{\left({L_{o}}^{*}-L^{*}\right)}^{2}+{\left({a_{o}}^{*}-a^{*}\right)}^{2}+{\left({b_{o}}^{*}-b^{*}\right)}^{2}}$$

where *L* ^*^ ranges from 0 (black) to 100 (white), positive *a* ^*^ denotes red and negative *a* ^*^ green, positive *b* ^*^ yellow and negative *b* ^*^ blue; the subscript 0 refers to the fresh reference ($L_{o}=15.147\pm 0.896;a_{o}=2.179\;\pm 0.260;\;b_{o}=9.254\pm 0.296$).

For total flavonoid, total phenolic, and antioxidant assays, the extract was prepared by combining 2 g of sample with 20 mL of methanol (95%) and 20 mL of acetone (70%) in test tubes. The tubes were shaken in an orbital shaker for 30 min, followed by sonication for 30 min. The mixture was then centrifuged for 5 min, and the supernatant was filtered through quantitative filter paper. The filtrate was transferred to a 50‐mL volumetric flask and brought to volume with distilled water.

Total flavonoids were quantified by the method of Arvouet‐Grand et al. (1994), with minor modifications. Briefly, 150 µL of 2% (w v^−1^) AlCl_3_ in ethanol (100%) was mixed with 150 µL of extract and allowed to stand at room temperature for 1 h. Absorbance was then recorded at 420 nm, using distilled water as the blank. Results were expressed as milligrams of quercetin per 100 g of sample.

Total phenolics were determined using the Fast‐Blue assay (Medina 2011) with slight adaptations. Aliquots of 50 µL of extract, 200 µL of distilled water, 25 µL of Fast‐Blue reagent (0.1%), and 25 µL of NaOH (5%) were combined in each well. After 1.5 h of incubation in the dark, absorbance was read at 420 nm. Measurements were run in triplicate on a flat‐bottom 96‐well microplate reader (Biochrom EZ Read 2000). Results are reported as mg gallic acid equivalents (GAE) per 100 g dry basis.

Antioxidant activity was assessed by ABTS·⁺ radical scavenging. The radical solution was prepared by reacting 7 mmol L^−1^ 2,2‐azinobis‐(3‐ethylbenzothiazoline‐6‐sulfonic acid) diammonium salt with 2.45 mmol L^−1^ potassium persulfate and allowing the mixture to stand at room temperature for 16 h. The resulting stock was diluted with ethanol to an absorbance of 0.70 ± 0.05 at 734 nm. Aliquots of 3 µL of extract were placed in the wells of a flat‐bottom 96‐well plate (Biochrom EZ Read 2000), followed by 297 µL of the ABTS·⁺ solution. After 6 min of reaction in the dark, absorbance was read at 734 nm. Antioxidant activity was expressed as ABTS reduction (%) according to Equation (22) (Auzanneau et al. 2018).

(22) $$\%\mathrm{ABT}S_{\mathrm{reduction}}=\left(100-\frac{\mathrm{AB}S_{\mathrm{control}}-\mathrm{AB}S_{\mathrm{sample}}}{\mathrm{AB}S_{\mathrm{control}}}\right)100$$

where $\mathrm{AB}S_{\mathrm{control}}\;$ and $\mathrm{AB}S_{\mathrm{sample}}$ are the absorbances of the control and the sample, respectively.

### 2.4 Oil Extraction and Yield

Fresh and dried seeds from each treatment were ground in a blender to increase the solvent–particle contact area. The ground material was then subjected to Soxhlet extraction with hexane 100% as the solvent (Fonseca et al. 2023). Papaya‐seed oil yield (PSOY) was calculated using Equation (23) (Chielle et al. 2016b):

(23) $$\mathrm{ROSM}=\frac{P_{o}}{P_{s}-\left(\frac{P_{s}X}{100}\right)}100$$

where ROSM is the oil yield (%), $P_{o}$ the mass of extracted oil (g), $P_{s}$ the mass of PS (g), and X the seed moisture content (g 100 g^−1^, dry basis).

### 2.5 Structural Analysis

Internal microstructure was examined by scanning electron microscopy (SEM). Samples were flash‐frozen in liquid nitrogen and fixed for 24 h at 4°C in a modified Karnovsky solution (2.5% glutaraldehyde + 2.5% formaldehyde in 0.05 M sodium cacodylate buffer, pH 7.2). They were then rinsed three times (10 min each) in the same buffer and dehydrated through an acetone series (25%, 50%, 75%, and 90%, 10 min each) followed by three 10 min washes in 100% acetone. Dehydrated specimens were critical‐point dried (CPD 030, Bal‐Tec), mounted on aluminum stubs with carbon double‐sided tape, and sputter‐coated with gold (SCD 050, Bal‐Tec). Imaging of fresh and dried samples was performed on a TESCAN CLARA UHR‐SEM (TESCAN Ltd., Kohoutovice, Czech Republic) at 6 kV, 69.6 µm working distance, and 6 k× magnification. Cell‐wall thickness was measured in ImageJ, taking three samples per treatment and five random points per sample (Santos et al. 2024; Silveira et al. 2024).

### 2.6 Statistical Analysis

Data were subjected to analysis of variance (ANOVA). Significant effects were followed by Tukey test for multiple comparisons, whereas each dried treatment was individually contrasted with the fresh‐seed control by Dunnett test. All analyses were performed at a 5% significance level using Statistica 8.0 (StatSoft Inc., Tulsa, OK, USA).

## 3 Results and Discussion

### 3.1 Drying

**FIGURE 1:**
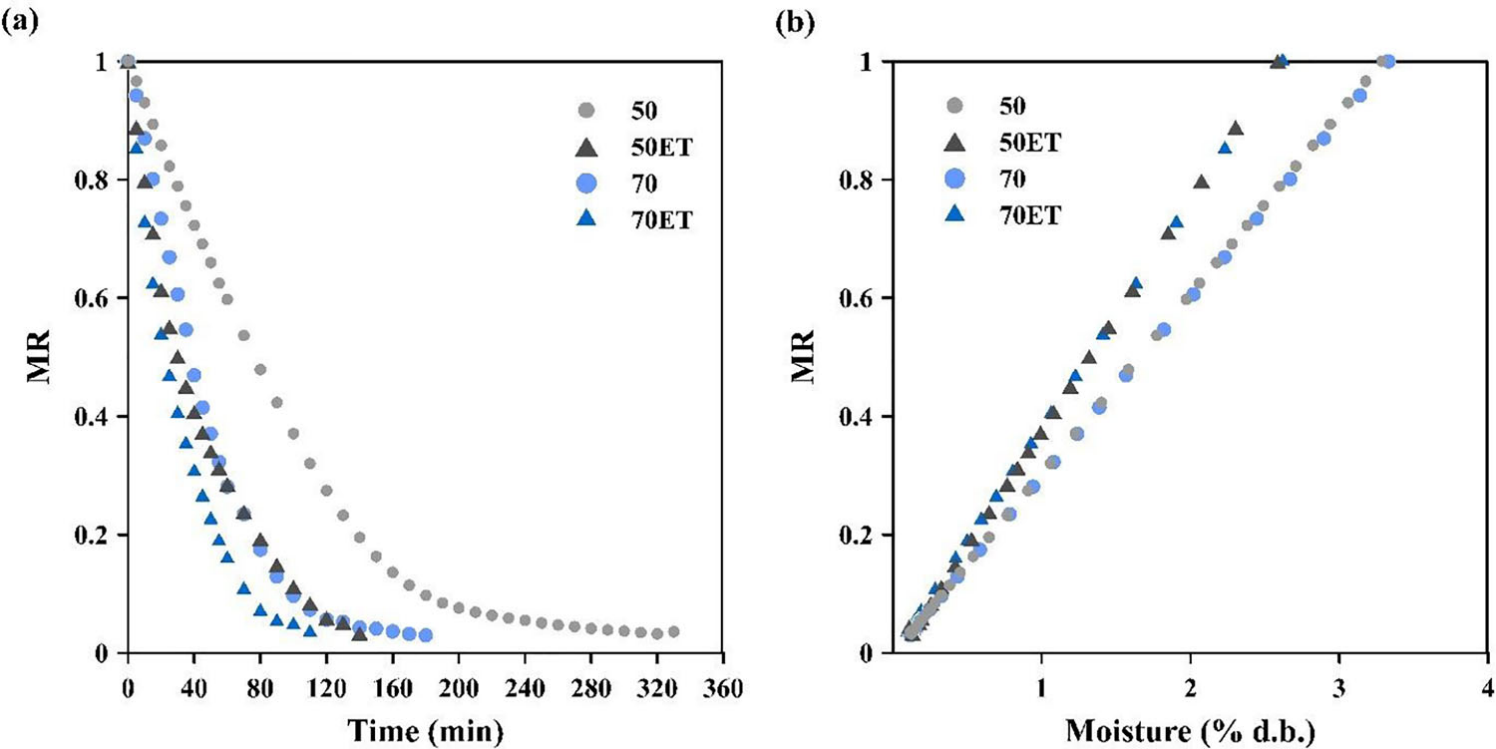
(a) Dimensionless moisture ratio (MR) as a function of drying time and (b) its relationship with moisture content on a dry basis, for papaya seeds dried at 50°C and 70°C. ET indicates ethanol pretreatment.

Figure 1 shows the evolution of the dimensionless moisture ratio (MR) of PS with drying at two temperatures, with and without ET. Final moisture content was set to 10.502 ± 0.283 (w.b.) in all cases.

Temperature presented a direct influence on moisture reduction apart from the ET use. Drying time was consequently reduced by about 45% by increasing the temperature from 50°C to 70°C. The higher the thermal energy applied, the higher the moisture internal diffusion and vapor pressure. Higher temperatures lower both internal and external resistances to moisture removal by increasing water‐molecule mobility (Junqueira et al. 2021).

ET also reduced drying time. Relative to untreated seeds, ethanol reduced drying time by 39% at 70°C and by 55% at 50°C. This behavior can be explained partly by the Marangoni effect: The surface‐tension difference between ethanol and water promotes preferential evaporation of ethanol, generating interfacial tension gradients that drive residual water toward the surface. The benefit is greater at lower temperatures, where solvent evaporation is slower and ethanol penetrates deeper into the matrix (Gomes et al. 2022). The stronger interaction intensifies the Marangoni effect, redistributing moisture and facilitating its outward flow. Similar findings have been reported for bananas (Corrêa et al. 2012), strawberries (Macedo et al. 2023), white and red dragon‐fruit (Macedo et al. 2021), taioba leaves (Junqueira et al. 2021), yacon (Silveira et al. 2024), and acerola seeds (Santos et al. 2024).

**FIGURE 2:**
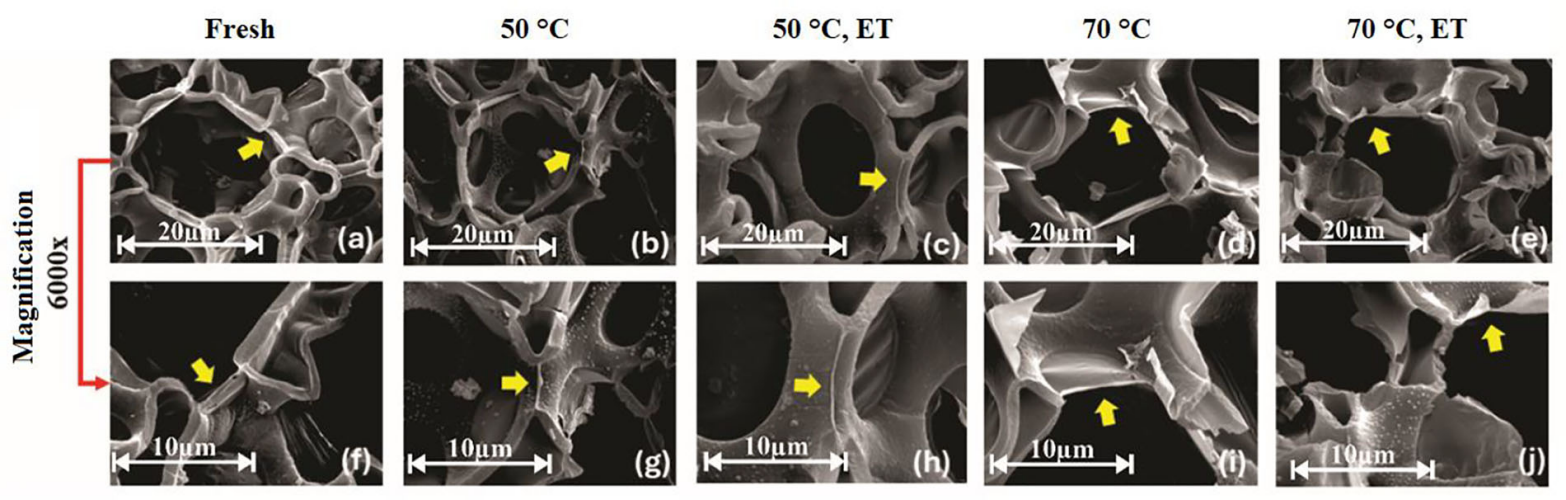
Scanning electron micrographs of papaya seeds: (a–e) at 6000× magnification and (f–j) at 12 000× magnification. Sample conditions are indicated in the figure (Fresh, 50°C, 50°C ET, 70°C, and 70°C ET). ET denotes ethanol pretreatment.

Thus, temperature and ethanol act synergistically: Higher temperatures hasten evaporation and moisture diffusion, whereas ethanol enhances moisture diffusion, together reducing the drying time. Ethanol can also cause morphological changes, altering structure and thinning cell walls, which raise permeability and facilitate the release of larger molecules (Gomes et al. 2022; Nogueira et al. 2023; Santos et al. 2024; Silveira et al. 2024). Figure 2 confirms that overall cell shape was largely preserved after drying; the surface remained porous, as previously reported by Chielle et al. (2016b). Seeds dried at 50°C (Figure 2b,c) retained more uniform, intact walls, whereas those dried at 70°C (Figure 2d,e) displayed thinner, brittle walls, evidence of greater thermal stress. All dried samples showed more cellular degradation than fresh seeds (Figure 2a). Ethanol‐treated samples (Figure 2c,f) exhibited noticeably rougher surfaces, probably due to partial matrix dissolution, which in turn eases moisture removal, an effect also observed in yacon (Silveira et al. 2024) and acerola seeds (Santos et al. 2024).

Previous SEM data (Santos et al. 2009) reported significant morpho‐anatomical changes in heat‐treated PS, including reduced cell‐wall thickness and altered morphology, features that influence cellular integrity and water‐holding capacity during drying. These findings are corroborated by the wall‐thickness measurements highlighted with yellow arrows in Figure 2 and summarized in Table 5. The cell‐wall thickness was reduced by 33.93% at 50°C and 63.62% at 70°C compared with fresh seeds. Ethanol intensified the effect, lowering wall thickness by 42.32% at 50°C and 74.73% at 70°C. The reduction is attributed to loss of turgor as water and solutes leave the tissue and to increased wall permeability caused by ethanol. Despite thinning, no wall rupture was observed, as noted by Silveira et al. (2024) in yacon drying. It should be noted that the variability of the measurements (Table 5) contributes to increased statistical deviations and errors, which is inherent to the heterogeneity of biological materials. Nevertheless, ethanol improves cellular microstructure and shortens processing time, as previously reported by Silveira et al. (2024) and Santos et al. (2024).

**TABLE 5:** Cell wall thickness of papaya seeds for fresh and convective dried (50°C and 70°C).

Treatment	Thickness (µm)
Fresh material	451.00 ± 157.69
50	298.00 ± 146.89^ab*^
50ET	260.00 ± 72.25^bc*^
70	164.00 ± 106.41^bc*^
70ET	114.00 ± 60.53^c*^

Abbreviation: ET, ethanol treatment.

### 3.2 Energy Consumption

**FIGURE 3:**
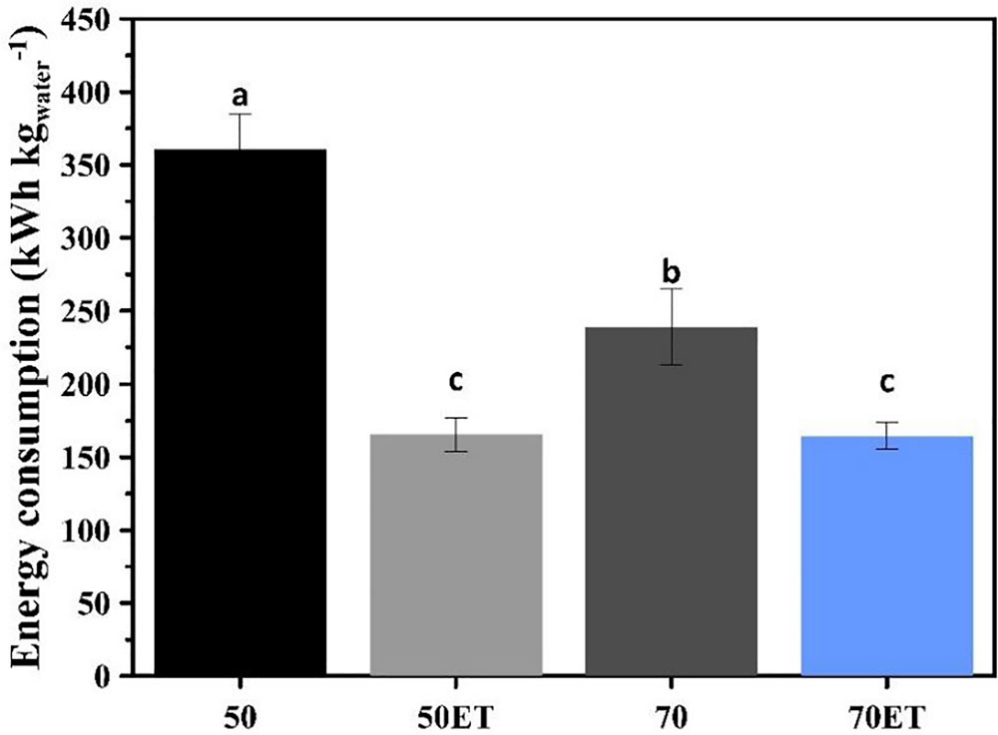
Energy demand under different drying conditions. Different letters indicate significant differences (Tukey's test, *p* < 0.05) between ethanol‐pretreated (ET) samples and control samples (without pretreatment).

Figure 3 compares the specific energy consumption for drying PS under the different conditions. Drying at 50°C without pretreatment was the condition with higher energy demand, 360.3567 kWh kg^−1^, owing to the lower evaporation rate at this temperature. As widely reported, lower temperatures prolong drying time and thus increase the energy required to sustain operation (Silveira et al. 2024). At 70°C, 238.8496 kWh kg^−1^ was consumed. The higher the temperature, the shorter the process with lower energy consumption.

When ET was applied, energy consumption dropped sharply to 165.5197 kWh kg^−1^ and 164.4951 kWh kg^−1^, at 50°C and 70°C, respectively, with no significant difference between the energy required for both temperatures. This is associated with higher moisture removal. Similar reductions were observed for yacon slices (Silveira et al. 2024) and papaya pulp (Cruz et al. 2025).

### 3.3 Mathematical Modeling of Drying Kinetics

**FIGURE 4:**
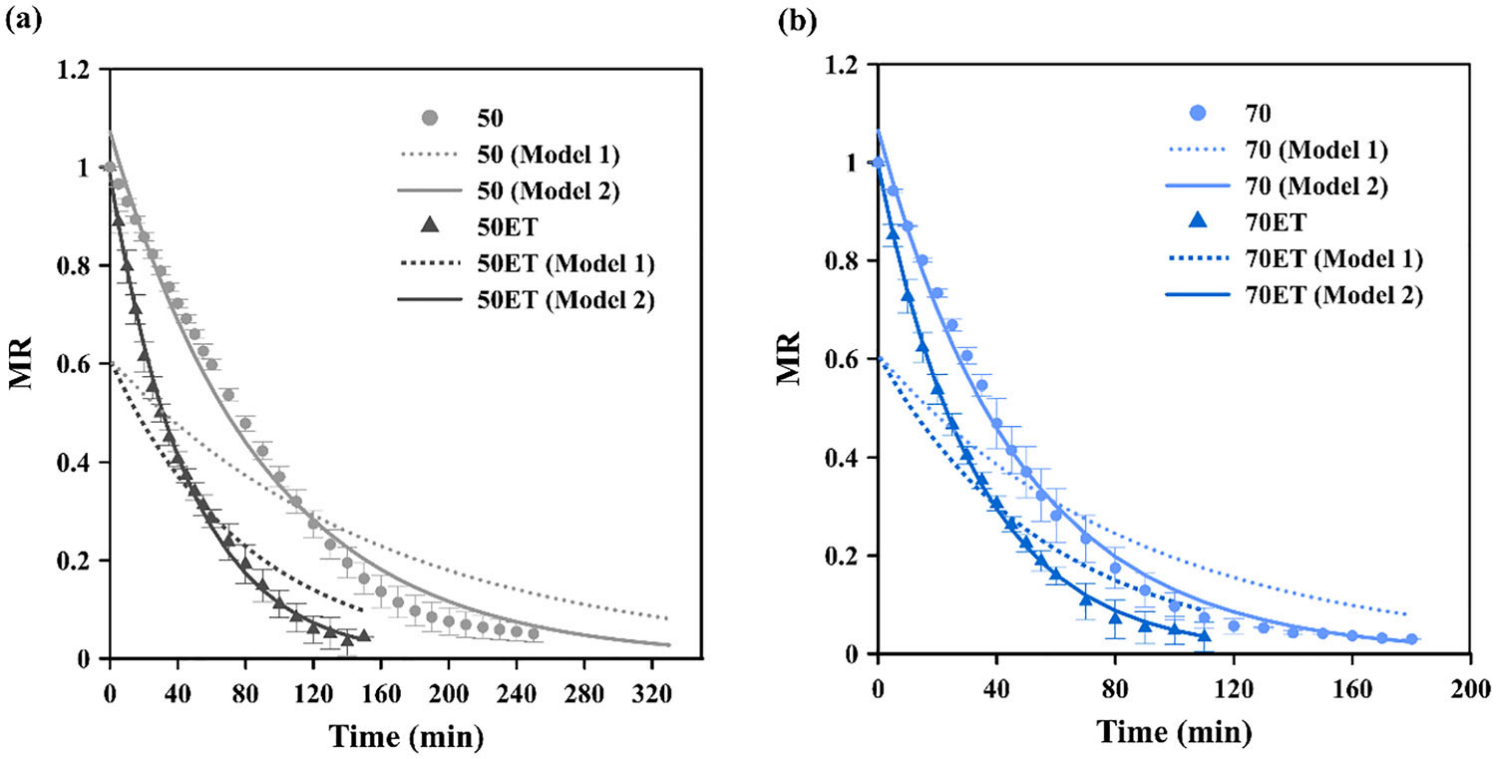
Moisture reduction profiles at 40°C (a) and 60°C (b), where symbols represent experimental observations and lines correspond to predicted values.

The fit of the experimental moisture‐ratio data for PS to the exponential expressions of Model 1 and Model 2 under the various drying conditions is shown in Figure 4, and the corresponding fitting parameters are listed in Table 6.

**TABLE 6:** Model fitting results: Coefficient of determination (*R* ^2^) and sum of squared errors (SSE).

Treatment	Model 1		Model 2	
	*R* ^2^	SSE	*R* ^2^	SSE
50	0.7086	1.2822	0.9908	0.0404
50ET	0.7503	1.0714	0.9979	0.0010
70	0.7149	0.7189	0.9934	0.0165
70ET	0.7508	0.3604	0.9992	0.0011

Abbreviation: ET, ethanol treatment.

The results (Figure 4 and Table 6) indicate that Model 2 shows a higher ability to describe the experimental data and to provide more accurate predictions than Model 1. Table 7 reports the parameters of this superior fit together with the effective moisture‐diffusivity values. Parameter *a* (Table 7) is close to 1, well removed from 6/π^2^, implying that mass transfer during papaya‐seed drying is not governed solely by internal diffusion (Nguyen et al. 2023) but by external and/or mixed resistance.

**TABLE 7:** Fitted parameters of the exponential model applied to the drying kinetics of papaya seeds, along with the corresponding values of effective moisture diffusivity.

Treatment	*a*	*b* (min^−1^)	*D*_eff 1_ (m^2^ s^−1^)	*Bi*_m_	*D*_eff 2_ (m^2^ s^−1^)
50	1.07160	0.00019	6.57134x10^−10^	−2.50049	—
50ET	0.98432	0.00036	1.28373 x10^−9^	0.851318	1.28266x10^−6^
70	1.06549	0.00035	1.24599 x10^−9^	−2.30823	—
70ET	0.99396	0.00051	1.79482 x10^−10^	0.400064	2.62633x10^−6^

Abbreviations: *Bi*_m_, the mass Biot number; D_eff1_ and D_eff2_, the diffusion coefficients that account for internal and mixed resistance domains, respectively; ET, ethanol treatment. The letters *a* and *b* are model parameters.

The controlling resistance regime was evaluated with the $Bi_{m}$ (Nguyen et al. 2023), also shown in Table 7. For seeds without the ET, $Bi_{m}$ < 0.1 denotes predominance of external mass resistance, in agreement with Junqueira et al. (2024). For the ET samples, $Bi_{m}$ > 0.1 reveals a mixed regime in which both internal diffusion and external resistance contribute to moisture removal, consistent with Bualuang et al. (2019) and Santos et al. (2024).

The effective diffusivity (*D*_eff_) increased with both ET and temperature, as likewise reported by Junqueira et al. (2021) and Macedo et al. (2023). Drying without ET is thus limited by external mass resistance, associated with the convective mass‐transfer coefficient, whereas drying with ET operates under mixed control. ET raises *D*_eff_ by thinning the cell wall and improving contact between the seed surface and the drying air, thereby facilitating moisture migration to the surface (Rojas et al. 2021).

### 3.4 Quality Parameters

#### 3.4.1 Water Activity (*a*_w_)

The *a*_w_ of the dried samples ranged from 0.25 ± 0.03 to 0.33 ± 0.02, with no significant differences among the dried treatments (50, 50ET, 70, and 70ET) because the dried samples presented similar final moisture content with no additional modification on the interactions between the biological material and moisture due to the ET. Lowering water activity in the dried seeds to 0.25–0.33 confirms the efficiency of the treatments, as values below 0.60 are sufficient to inhibit microbial growth and extend shelf life (Toscano et al. 2019). Although the drying temperatures did not differ statistically, strict thermal control remains essential to preserve the physicochemical integrity of the seeds, particularly for matrices rich in bioactive compounds (Mujumdar 2006). Consequently, the drying methods applied, including ET, proved effective in stabilizing the seeds and prolonging their shelf life.

#### 3.4.2 Color Parameters

**FIGURE 5:**
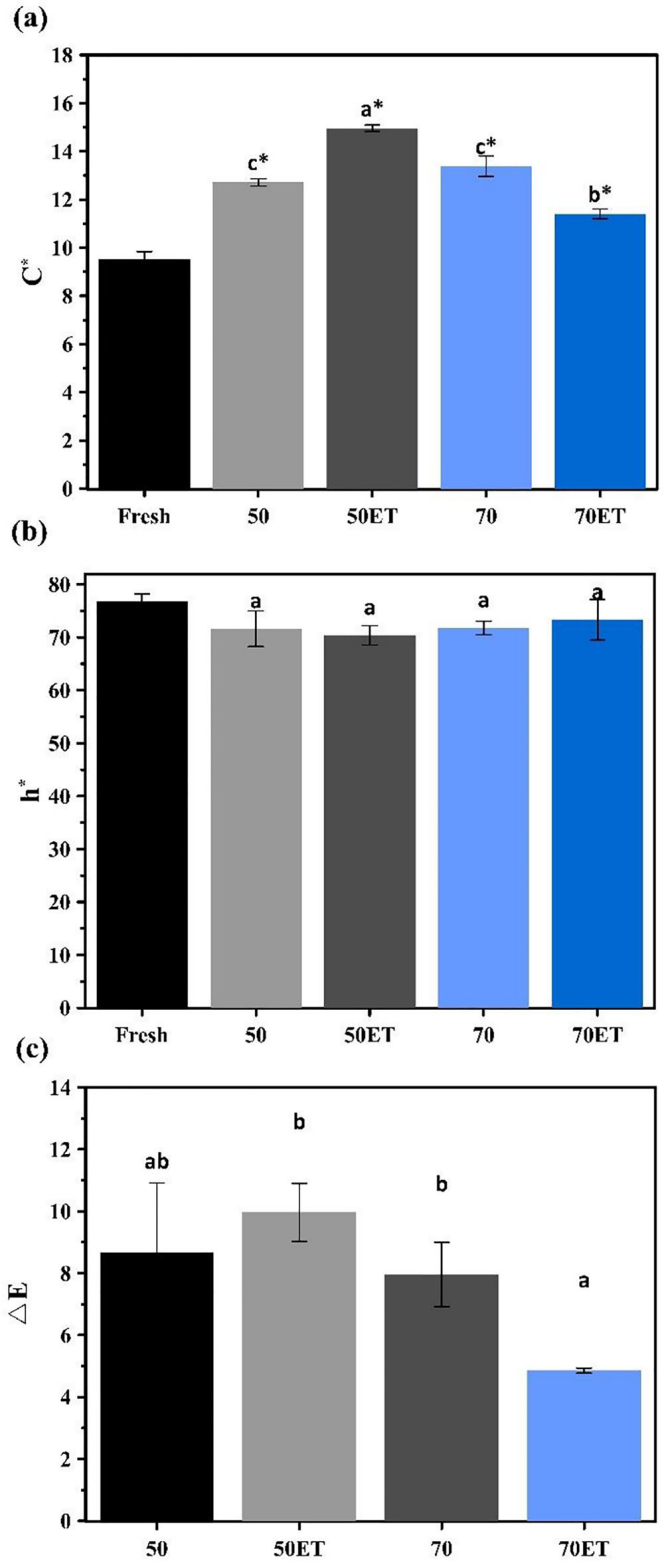
Colorimetric parameters of papaya seeds (a) C^*^, (b) h^*^, and (c) Δ*E*. Equal letters denote no significant difference among drying treatments (Tukey's test, *p* < 0.05), while asterisk indicate significant differences compared to the fresh sample (Dunnett'test, *p* < 0.05).

Chroma (*C* ^*^, Figure 5d), which represents color saturation, peaked at 50ET (*C* ^*^ = 14.95), indicating a more vivid appearance preferred by consumers; the lowest *C* ^*^ was found in the fresh control (9.51). These findings confirm that selected temperatures combined with ethanol can intensify saturation and enhance visual quality (Cruz et al. 2025). Hue angle (*h* ^*^, Figure 5e) ranged from 70° to 76°, denoting a predominantly yellow‐green hue, and did not differ significantly among dried treatments.

Total color difference (∆*E*) showed that 50ET produced the greatest perceptible change (∆*E* = 9.96), whereas 70ET exhibited the smallest (∆*E* = 4.85). Thus, moderate temperatures together with ET markedly influence seed color, either preserving or controllably modifying natural pigments (Macedo et al. 2023; Santos et al. 2024).

#### 3.4.3 Bioactive Compounds

**FIGURE 6:**
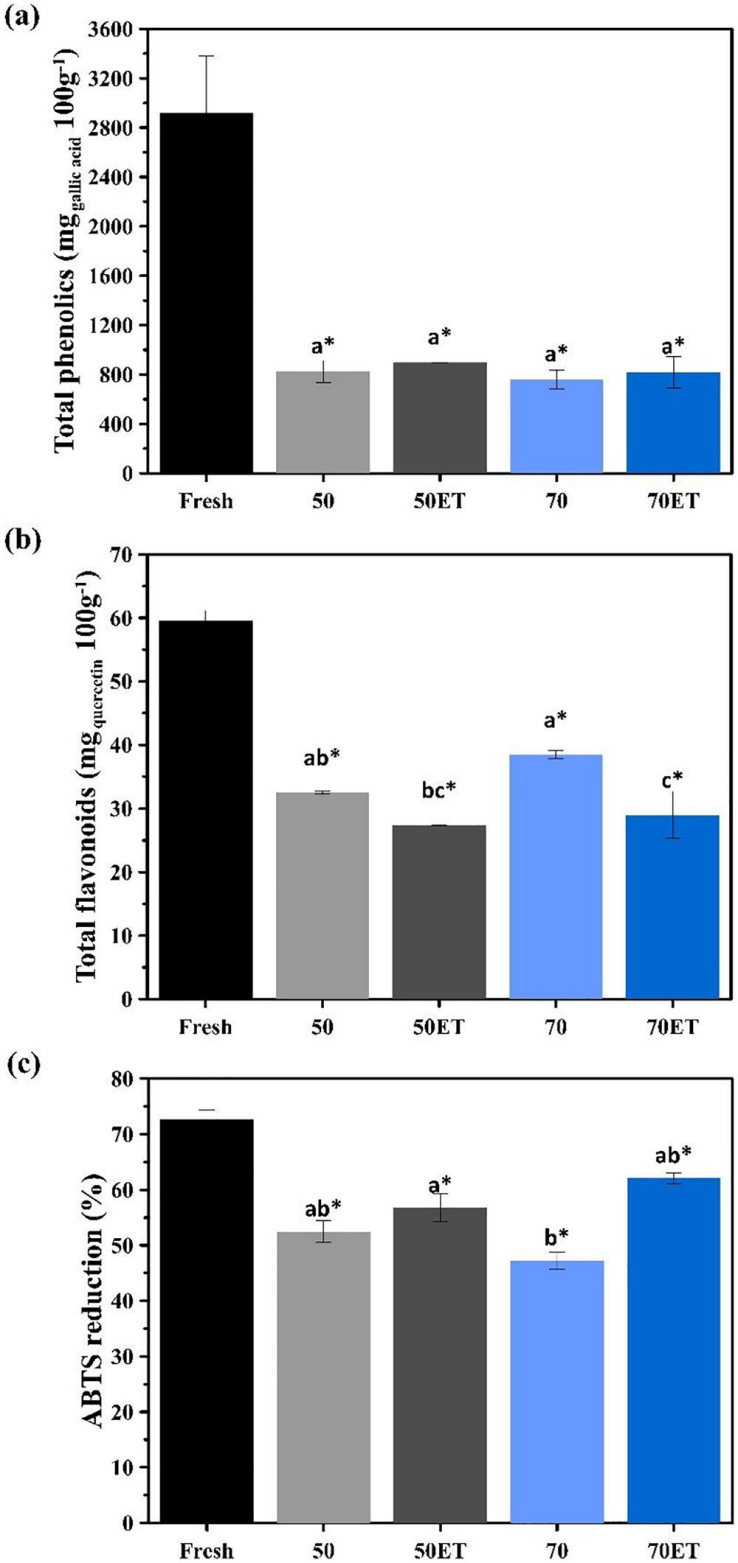
Representation of bioactive compounds (a) total phenolics (TPC), (b) flavonoids, and (c) antioxidant activity measured by the ABTS reduction method. Equal letters indicate no significant difference between treatments (Tukey's test, *p* < 0.05). The asterisk indicates a significant difference between the residues subjected to the drying treatment and the fresh residues (Dunnett's test, *p* < 0.05).

With respect to total phenolic content (TPC, Figure 6a), all dried samples differed significantly from the fresh residue; however, no statistical difference was detected among the drying treatments. Such differences between the fresh and dried samples are commonly reported in the literature, since drying may simultaneously lead to phenolic losses and thermal degradation, while cell wall disruption can also release vacuolar phenolics, either enhancing their extractability or, under certain conditions, favoring further degradation (Junqueira et al. 2021; Nogueira et al. 2023).

Flavonoid analysis (Figure 6b) likewise revealed significant differences between the fresh sample and all dried samples. Because flavonoids are thermosensitive, prolonged exposure to high temperatures accelerates their degradation; thus, the observed levels reflect the time/temperature combination applied (Castro et al. 2017). Fujita et al. (2013) reported that higher drying temperatures can be less destructive to flavonoids if accompanied by shorter drying times, as observed for spray‐dried camu‐camu pulp. In the ethanol pretreated samples, however, flavonoid content was lower. Studies indicate that ethanol can interact directly with flavonoids, rendering them more susceptible to oxidation and degradation (Chávez‐González et al. 2020). Moreover, ET may alter cell structure, exposing internal compounds to thermal and oxidative stress during drying (Cruz et al. 2025). This combination explains the contrast between treated (70ET) and untreated (70) seeds, the ethanol treatment resulting in poorer flavonoid retention.

Antioxidant activity assessed by ABTS reduction (Figure 6c) decreased after thermal treatment, consistent with the loss of bioactive compounds (Castro et al. 2018). Nevertheless, no significant difference was observed between paired treatments (50 vs. 50ET, 70 vs. 70ET).

#### 3.4.4 Oil Yield

Table 8 shows that no significant differences were observed in oil yield between fresh seeds and those subjected to different drying treatments, with or without ET. These results indicate that water removal, under the evaluated conditions, did not compromise the amount of extractable oil.

**TABLE 8:** Oil yield of fresh papaya seeds and seeds subjected to different treatments.

Treatment	ROSM (d.b.) (%)
Fresh	9.197 ± 1.665
50	11.135 ± 1.522^a^
50ET	10.457 ± 1.168^a^
70	10.415 ± 1.789^a^
70ET	13.809 ± 0.287^a^

Abbreviation: ET, ethanol treatment.

Identical letters denote no significant difference among treatments according to Tukey test (*p* < 0.05).

According to Chielle et al. (2016a), lower drying temperatures slow down water evaporation and therefore induce fewer structural changes, but prolong exposure to heat. In contrast, higher temperatures reduce processing time but may promote structural and chemical modifications, such as lipid oxidation, protein denaturation, and carbohydrate degradation. In the present study, however, such alterations did not significantly affect lipid extraction, suggesting that the oil present in PS exhibits stability under the tested conditions.

Although ethanol is recognized as an efficient solvent for oil extraction, previous studies have demonstrated that short contact times are not sufficient to promote relevant lipid extraction (Toda et al. 2016; Sánchez et al. 2019). Thus, the ET applied in this work did not interfere with the final oil yield, reinforcing its feasibility as a drying intensification strategy without losses in the lipid fraction.

## 4 Conclusion

This study examined the effect of ET on convective drying of PS. Raising the air temperature from 50°C to 70°C shortened the total drying time by up to 45%, with further drying time reduction up to 55% by applying ET at 50°C, reducing also the energy consumption. SEM revealed a greater surface roughness and thinner cell walls in ethanol‐treated seeds, features that increased permeability and facilitated water removal. Mathematical modeling confirmed that the pretreatment shifted mass transfer to a mixed regime in which both internal diffusion and external resistance are relevant.

Drying largely preserved total phenolics and ABTS antioxidant activity, especially in ethanol‐treated samples, whereas flavonoids were more susceptible to degradation, underscoring the need to fine‐tune time/temperature conditions. Color analysis showed that drying at 50°C with ET best maintained brightness and pigment saturation.

Overall, combining ET with controlled temperatures optimized papaya‐seed drying, reducing processing time and energy use without compromising product quality. The findings support the use of PS as a sustainable ingredient for food and pharmaceutical applications and encourage further research into valorizing agricultural residues.

## Author Contributions

**Amanda Aparecida de Lima Santos**: conceptualization, investigation, writing–original draft, methodology, validation, visualization, writing–review and editing, project administration, formal analysis, software, data curation, supervision, resources. **Maria Eduarda Teixeira**: data curation, formal analysis, investigation, methodology, resources, software, validation, visualization, writing–original draft. **Paula Giarolla Silveira**: conceptualization, investigation, methodology, software, writing–review and editing. **Jefferson Luiz Gomes Corrêa**: funding acquisition, writing–review and editing, supervision, investigation.

## Funding

This study was supported by the Conselho Nacional de Desenvolvimento Científico e Tecnológico (CNPq) (314191/2021‐6), Coordenação de Aperfeiçoamento de Pessoal de Nível Superior (CAPES) (88887.705821/2022‐0), and Fundação de Amparo à Pesquisa do Estado de Minas Gerais (FAPEMIG) (APQ‐01076‐24).

## Conflicts of Interest

The authors declare no conflicts of interest.

## Data Availability

The data will be made available on request.
